# Headache symptoms from migraine patients with and without aura through structure-validated self-reports

**DOI:** 10.1186/s12883-017-0973-4

**Published:** 2017-10-12

**Authors:** Jiawei Wang, Bingren Zhang, Chanchan Shen, Jinhua Zhang, Wei Wang

**Affiliations:** 1 Department of Clinical Psychology and Psychiatry/School of Public Health, Zhejiang University College of Medicine, Hangzhou, China; 2 Department of Neurology, Zhejiang Provincial People’s Hospital, Hangzhou, China

**Keywords:** Factor analysis, Headache symptom, Migraine with aura, Migraine without aura, Patients’ self-reports

## Abstract

**Background:**

Headache symptoms self-reported by migraine patients are largely congruent with the clinician-used diagnostic criteria, but not always so. Patients’ self-reports of headache symptoms might offer additional clues to characterize migraine with (MA) and without (MO) aura more precisely.

**Methods:**

Firstly, we invited 324 participants with a life-long headache attack to answer an item-matrix measuring symptoms of primary headaches, then we performed both exploratory and confirmatory factor analyses to their answers and refined a headache symptom questionnaire. Secondly, we applied this questionnaire to 28 MA and 52 MO patients.

**Results:**

In participants with a life-long headache, we refined a 27-item, structure-validated headache symptom questionnaire, with four factors (scales) namely the Somatic /Aura Symptoms, Gastrointestinal and Autonomic Symptoms, Tightness and Location Features, and Prodromal/Aggravating Symptoms. Further, we found that MA patients reported higher than did MO patients on the Somatic/Aura Symptoms and Tightness and Location Features scales.

**Conclusions:**

Compared to MO, MA was conferred with more prominent tightness and location features besides its higher somatic or aura symptoms. Patients’ self-reports of headache symptoms might offer more clues to distinguish two types of migraine besides their clinician-defined criteria.

## Background

Migraine is a common disabling primary headache disorder with head pain and autonomic and neurological symptoms [1]. Its diagnosis relies largely on the symptomatology due to the lack of clearly detectable biological markers and explicit radiological features [2]. It is then actually among the most under-recognized and under-treated neurological disorders [3]. The generally accepted diagnostic criteria for primary headaches are those published by the International Headache Society, such as the International Classification of Headache Disorders [4]. These criteria are comprehensive but still need to be further improved. Moreover, the effective application of these criteria requires trained professionals with experience and knowledge and it is not feasible to take a physical exam and medical history in large population-based studies. Besides, physicians’ diagnoses depend more on their clinical experience and inconsistent interpretations of these criteria in clinical practices, to some extent that personal description of patients has been neglected.

Besides the overlap of neurological symptoms and nonmutual exclusivity of aura symptoms, there has been long-standing controversy about obligatory characteristics for migraine with (MA) and migraine without aura (MO). According to the definition of the International Headache Society, migraine aura is the reversible focal neurological symptoms that arise before or during a migraine attack. However, clinicians have found that aura might occur before or during a migraine attack, occur without any associated headache, or occur with many other types of headache [5–7]. In addition, MO and MA displayed different clinical symptom patterns during pregnancy [8]. Whether the two migraine types are distinct entities in etiology and clinical pattern or they just differ in degree rather than in pathophysiology remains unclear [9].

Further, the reduced parasympathetic activity with sympathetic predominance, and the increased frequency of anxiety and depressive symptoms were found in migraine, especially in MA [10, 11]. One pharmacological study has shown that MA attacks were more severe and the treatment was less effective [12]. One functional magnetic resonance imaging study [13] has demonstrated that there were abnormalities in the cortical and subcortical pain processing networks in MO rather than in MA; during the interictal period, the functional connection between the occipital lobe and the frontal insula of MA was reduced than that of MO. Tedeschi et al. [14] also have found that the functional connection between gyrus lingualis and visual cortex was enhanced during interictal period, which might imply that the central sensitization effect and cortical hyperactivity is a unique pathogenesis of MA [15, 16].

On the other hand, there has been an increasing academic interest in investigating the clinical, epidemiological, and genetic problems of primary headaches, especially about the most common ones – the tension-type headache and migraine. In most studies of migraine, the methods of data acquisition include personal interview, telephone interview and self-administered questionnaire reports [3]. Symptoms reported by patients using a structure-validated symptom questionnaire are limited, most symptom studies however, were from the hospital-based medical records, professional physician interviews in clinics [17]. The distinction between these methods is not always as straight forward as it may be [3]. Differences in screening procedure (e.g., wording differences) may have significant influences on the estimation of headache disorders [2]. The self-reported questionnaire, which can be easily implemented to large samples, is an effective measure to access many diseases and explore constructs that would be difficult to obtain through behavioral or physiological measures. Fortunately, in a migraine study of Women’s Health Study sub-cohort, the self-reported migraine and the migraine classified based on the International Classification of Headache Disorders-II revealed a satisfactory agreement [18].

Thus, in the current study, we have invited a group of headache patients to report their complaints of before, during and after a headache attack, since in clinics, a complete migraine includes the prodromal, aura, and headache phases [19]. Patients were also invited to report their knowledge about headache, treatment-seeking behavior, and family history of headache, which might serve as the contextual headache information. Based on the reported symptoms and the statements of the International Classification of Headache Disorders, we developed an item-MATRIX measuring symptoms of primary headaches. The purposes of the present study were (1) to obtain a structure-validated headache symptom questionnaire from the item-MATRIX, and (2) to look for the different aspects of headache symptom between MA and MO through the questionnaire self-reporting. We have hypothesized that both MA and MO patients report their headache symptoms fitting to a time sequence, and MA patients report more intensified headache symptoms than MO patients do apart from the aura.

## Methods

**Fig. 1:**
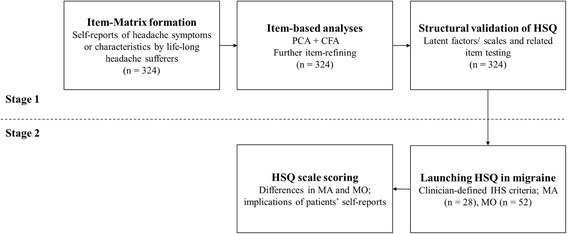
Study flowchart

The present study contained two stages (see Fig. 1). Firstly, we used the exploratory factor analysis and confirmatory factor analysis on a headache item-MATRIX to develop a structure-validated headache symptom questionnaire, following the scale development guidelines proposed by DeVellis [20]. Secondly, we applied the headache symptom questionnaire to both MA and MO patients.

### Questionnaire development

#### Participants

In total, 324 participants (131 men, age range 16–65 years, mean age 22.16 years ±7.87 S.D.; 193 women, age range 14–75, mean age 24.32 ± 11.26) were recruited from undergraduate students, medical staff-members and clinical out-patients. All participants had a complaint of headache during their life-long period, but did not suffer from any psychiatric or neurological (including neuroinflammatory) disorders, had no prior history of head injury, no alcohol or tobacco abuse, and no substance abuse. The study was approved by a local Ethics Committee, and all participants gave their written informed consents to participate.

#### Procedure

The headache item-MATRIX included the qualitative dimensions of headache: the headache characteristics, headache-related symptoms, aura symptoms, triggers, aggravating and relieving factors, health-seeking behaviors, etc. Considering the importance of every components and dimensions, 71 items were carefully constructed using the statements of the interviewees. The Likert rating scales, 1 - very unlike me, 2 - moderately unlike me, 3 - somewhat like and unlike me, 4 - moderately like me, 5- very like me, were chosen for the questionnaire.

#### Statistical analysis

The answers to the 71 items from the 324 subjects were submitted to a principal component analysis, using the Predictive Analytics Software Statistics, Release Version 18.0.0 (SPSS Inc., 2009, Chicago, IL). The factor loadings were rotated orthogonally using the varimax normalized method. Items which were loaded less heavily (loading ≤ .40) on a target factor, or cross-loaded heavily (cross-loading ≥ .35) on more than one factor were removed from subsequent analyses one-by-one. The procedure continued until no further item was needed to be removed. Afterwards, based on the latent factors, the fit of the structural equation modeling was evaluated by the confirmatory factor analysis using Analysis of Moment Structures (AMOS) version 17.0 (AMOS Development Corp., 2008, Crawfordville, FL). We used the following parameters to identify the model fit: the χ^2^/df, the goodness of fit index (GFI), the adjusted goodness of fit index (AGFI), the comparative fit index (CFI), the Tucker-Lewis index (TLI), the root mean square error of approximation (RMSEA), and the standardized root mean square residual (SRMSR). When the optimal model fit was established, the headache symptom questionnaire was developed based on the emerged factors (scales) and their high-loading items. The internal reliability (the Cronbach alpha coefficient) of each scale was then calculated. After both exploratory and confirmatory factor analyses, the structure-validated questionnaire was formed and then used in the second stage of the study.

### Application of the structure-validated questionnaire in migraine

#### Participants

Afterwards, the questionnaire was tested in migraine patients. Altogether, we invited 28 MA (code 1.2., 14 men, age range 16–65 years, mean age 19.43 years ±1.62; 14 women, age range 18–68, mean age 23.81 ± 10.00), and 52 MO (code 1.1., 7 men, age range 18–23, mean age 19.43 ± 1.62; 45 women, age range 14–64, mean age 23.81 ± 10.00) patients who were diagnosed according to the third beta edition of the International Classification of Headache Disorders (ICHD-3 beta; International Headache Society, 2013). There was no statistically significant difference in age distribution between two groups (*t* = −1.58, *p* = .12). Data about the headache attack frequency, headache attack duration, and headache intensity were also collected and used to confirm either MA or MO diagnosis. Patients were verified to receive no prophylactic therapy and had been drug-free for at least 24 h prior to the test. They did not suffer from any psychiatric or neurological (including neuroinflammatory) disorders, had no prior history of head injury, no alcohol or tobacco abuse, and no substance abuse. The study was approved by a local Ethics Committee, and all participants gave their written informed consents to participate.

#### Procedure

Patients were asked to complete the structure-validated questionnaire in a quiet room under supervision of a co-author of the paper (BZ).

#### Statistical analysis

The scale scores of the structure-validated questionnaire in MA and MO groups were submitted to two-way ANOVA (i.e., group x scale score) plus the independent Student *t* test. A *p* value inferior to .05 was considered to be significant.

## Results

### Questionnaire development

**Fig. 2:**
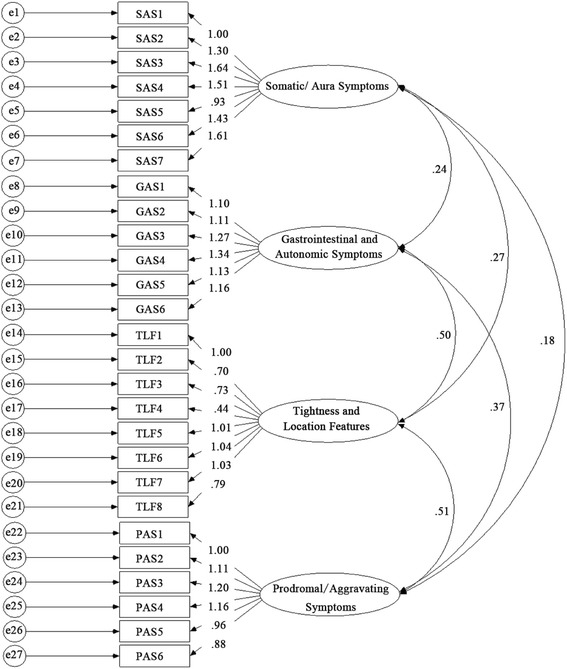
Standardized four-factor structure of the Headache Symptom Questionnaire in 324 participants

The principal component analysis extracted 20 factors with eigenvalues larger than 1.0. The screen plot and parallel analysis results suggested a seven-factor solution, and the first seven factors accounted for 41.90% of the total variance. When scrutinizing these latent factors and their items, four of which clearly described headache symptoms, and the remaining three described the knowledge about headache, treatment-seeking behavior, and family history of headache. Because the main purpose of the current design, we finally chose the four factors describing headache symptoms, which accounted for 37.03% of the total variance. Using the factor loading of .40 as a cutoff value, we constructed a fit modeling, with 27 items which were distributed in the four factors, and named the questionnaire as the Headache Symptom Questionnaire (HSQ, Fig. 2). In addition, the structural equation modeling confirmed that the four-factor modeling was a suitable solution (χ2/df = 2.00, GFI = .87, AGFI = .84, TLI = .87, CFI = .88, RMSEA = .057, SRMSR = .060).

The first HSQ factor with 7 items, e.g., “I felt my vision blurred when headache attacked”, and “I lost physical balance control when headache attacked”, is a mixture of somatic and aura symptom descriptions. The second factor with 6 items, e.g., “I had a poor appetite when headache attacked”, and “I looked pale when headache attacked”, narrates the gastrointestinal and autonomic symptoms. The third factor with 8 items, e.g., “I felt that my head was hooped by a ribbon when headache attacked”, and “Headache location migrated during my headache period”, describes the characteristics and location of headache. The fourth factor with 6 items, e.g., “I felt fatigue for a period before headache attacked”, describes prodromal or aggravating symptoms before or during headache attacks. The four HSQ factors were then named as the Somatic/Aura Symptoms (Factor 1, with an internal reliability of .79), the Gastrointestinal and Autonomic Symptoms (Factor 2, internal reliability .79), Tightness and Location Features (Factor 3, internal reliability, .81), and Prodromal/Aggravating Symptoms (Factor 4, internal reliability .80), respectively (Table 1).

**Table 1:** Factor loadings on the four factors in 324 participants

	Items	Factor			
		1	2	3	4
Somatic/Aura Symptoms	I felt my vision blurred when headache attacked.	**.71**	.10	.15	.10
	There were black spots in my vision when headache attacked.	**.70**	.16	.08	.12
	I talked less frequently when headache attacked.	**.68**	.15	.10	.00
	I lost physical balance control when headache attacked.	**.64**	.22	.11	.20
	I lost consciousness when headache attacked.	**.63**	.01	.24	−.16
	I was sensitive to strong sound when headache attacked.	**.49**	.22	.11	.17
	I felt abdominal pains when headache attacked.	**.49**	.23	.06	.24
Gastrointestinal and Autonomic Symptoms	I had a poor appetite when headache attacked.	.10	**.82**	.05	.08
	I looked pale when headache attacked.	.24	**.71**	.26	−.01
	I felt weak when headache attacked.	.16	**.60**	.18	.31
	I felt nausea when headache attacked.	.27	**.57**	.18	.18
	I was sensitive to strong light when headache attacked.	.21	**.47**	.31	.20
	I sweated a lot when headache attacked.	.34	**.41**	.19	.19
Tightness and Location Features	I felt that my head was hooped by a ribbon when headache attacked.	.22	.16	**.75**	.05
	I once felt that my head was hooped.	.07	.13	**.75**	.14
	I felt that my head was pressed by a big stone when headache attacked.	.31	.09	**.65**	.21
	I felt I was wearing a hat when headache attacked.	.28	.26	**.64**	.08
	My whole head was extremely painful when headache attacked.	−.03	.15	**.57**	.24
	Headache location migrated during my headache period.	.16	.09	**.41**	.10
	I felt throbbing over my head when headache attacked.	.00	.24	**.41**	.39
	My neck was also painful when headache attacked.	.33	−.05	**.41**	.36
Prodromal/Aggravating Symptoms	I felt fatigue for a period before headache attacked.	.11	.16	.08	**.78**
	I had difficulty in concentrating for a period before headache attacked.	.19	.15	.08	**.76**
	I yawned repetitively for a period before headache attacked.	.23	.05	−.03	**.72**
	I was often upset about my life or study/work.	−.01	.10	.26	**.56**
	I was easily irritated when headache attacked.	.11	.10	.30	**.55**
	Insomnia intensified my headache.	−.08	.20	.36	**.55**

Loadings higher than 0.40 are presented in bold for clarity

### Questionnaire application

The mean HSQ scale scores were significantly different between the two groups (F [1, 78] = 9.90, *p* = .002, mean square effect = 522.32). The *t* test showed that MA patients scored significantly higher than did MO patients on Somatic/Aura Symptoms (*p* < .01, 95% confidence interval (CI) = [5.76, 10.65]) and Tightness and Location Features (*p* = .01, 95% CI = [.89, 6.31]) (Table 2).

**Table 2:** Scale scores (mean ± S.D.) of the Headache Symptom Questionnaire in migraine with (*n* = 28) and without aura (*n* = 52) groups

	Migraine with aura	Migraine without aura
Somatic/Aura Symptoms	21.64 ± 5.60	13.44 ± 5.04*
Gastrointestinal and Autonomic Symptoms	20.11 ± 5.45	20.83 ± 4.43
Tightness and Location Features	28.25 ± 5.43	24.65 ± 6.00*
Prodromal/Aggravating Symptoms	23.71 ± 6.65	24.08 ± 5.48

*p < .05 vs Migraine with aura

## Discussion

After both exploratory and confirmatory factor analyses, we have developed a structure-validated Headache Symptom Questionnaire, with four scales: Somatic/Aura Symptoms, Gastrointestinal and Autonomic Symptoms, Tightness and Location Features, and Prodromal/Aggravating Symptoms. According to the self-reports, MA patients scored higher on the Somatic/Aura Symptoms and the Tightness and Location Features scales than MO patients did.

The first scale Somatic/Aura Symptoms, which describes vision, speech, motor control and some brainstem functions, is the complaints frequently reported from patients during the aura and headache phases [21]. It is stated that the aura normally disappears as the headache starts [4]. However, some scholars are wondering the role of aura in the initiation of headache when referring to its timing in relation to the headache and the prodromal symptoms [7, 22, 23]. Our results also suggest that the aura precedes or accompanies the onset of headache. Interestingly, Schürks et al. [18] have reported MA patients do not always report aura symptoms, while MO patients sometimes do so instead. The higher score of the Somatic/Aura Symptoms in our MA group suggest that it is the severity, frequency and complexity of aura symptoms that distinguishes the two types of migraine, rather than the simple presence of the aura.

The second scale embraces two aspects regarding gastrointestinal and autonomic symptoms, such as the nausea, pale face and hyperhidrosis. It is generally accepted that nausea is the main feature which characterizes migraine [4]. Previous studies do have demonstrated that the reduced parasympathetic activity with sympathetic predominance in patients with migraine [11].

The third scale describes the headache location and quality, which is included by the International Headache Society [4] to characterize tension-type headaches. However, patients with migraine, especially MA, also present bilateral head and neck pains [24]. Drummond [25] noted that tension-type headache patients had bilateral head pain but had few or no features of migraine. Our results showed that migraine had more complex manifestations than the headache criteria currently describe, which sends an appeal that headache specialists consider more about patients’ self-reports when diagnosing migraine. Furthermore, the higher score of the Tightness and Location Features scale we found was in line with that the MA manifestations varied more widely than those of MO did [26].

The fourth scale describes physical fatigues and psychiatric concerns related to the head pain. One population-based study found that some psychiatric comorbidities, particular mood and anxiety disorders, were common in migraine patients [27]. Other scholars suggested that the psychiatric comorbidities might be a risk factor for migraine chronification, i.e., for the progression from episodic form to chronic one [28]. Several studies have suggested that the prodromal dysfunctions might act as a primary trigger for a migraine attack [21, 29]. Our results indicate that the prodromal or aggravating symptoms reflecting the respective physical and psychiatric alterations reported by patients generally characterize the two types of migraine.

There were however, at least two limitations of our study design which should be considered. First, we did not enroll patients with other primary or secondary headaches. Second, our sample sizes of both MA and MO groups were relatively small. Future studies might include more headache controls and compare descriptions from patients’ self-reports and the clinical criteria.

## Conclusions

Through two series of study, we have demonstrated a structure-validated headache questionnaire for patients’ self-reports, and found that the two types of migraine might be distinguished by the Somatic/Aura Symptoms and the Tightness and Location Features scales. Patients’ self-reports of head pain symptoms might offer more clues to distinguish different headache types than do the clinician-defined criteria.

## Abbreviations

HSQ: The Headache Symptom Questionnaire
ICHD: The International Classification of Headache Disorders
MA: Migraine with aura
MO: Migraine without aura
